# Predictors of 30-day mortality among patients with stroke admitted at a tertiary teaching hospital in Northwestern Tanzania: A prospective cohort study

**DOI:** 10.3389/fneur.2022.1100477

**Published:** 2023-01-18

**Authors:** Sarah Shali Matuja, Gilbert Mlay, Fredrick Kalokola, Patrick Ngoya, Jemima Shindika, Lilian Andrew, Joshua Ngimbwa, Rashid Ali Ahmed, Basil Tumaini, Khuzeima Khanbhai, Reuben Mutagaywa, Mohamed Manji, Faheem Sheriff, Karim Mahawish

**Affiliations:** ^1^Department of Internal Medicine, Catholic University of Health and Allied Sciences, Mwanza, Tanzania; ^2^Department of Internal Medicine, Bugando Medical Centre, Mwanza, Tanzania; ^3^Department of Neurology, Harvard Medical School, Massachusetts General Hospital, Boston, MA, United States; ^4^Department of Internal Medicine, Muhimbili University of Health and Allied Sciences, Dar es Salaam, Tanzania; ^5^Department of Cardiology, Jakaya Kikwete Cardiac Institute, Dar es Salaam, Tanzania; ^6^Department of Neurology, Texas Tech University Health Sciences Center, Paul L. Foster School of Medicine, El Paso, TX, United States; ^7^Stroke Medicine Department, Counties Manukau Health, Auckland, New Zealand

**Keywords:** stroke, predictors, morbidity, mortality, Tanzania

## Abstract

**Background:**

Stroke is the second leading cause of death worldwide, with the highest mortality rates in low- to middle-income countries, particularly in sub-Saharan Africa. We aimed to investigate the predictors of 30-day mortality among patients with stroke admitted at a tertiary teaching hospital in Northwestern Tanzania.

**Methods:**

This cohort study recruited patients with the World Health Organization's clinical definition of stroke. Data were collected on baseline characteristics, the degree of neurological impairment at admission (measured using the National Institutes of Health Stroke Scale), imaging and electrocardiogram (ECG) findings, and post-stroke complications. The modified Rankin scale (mRS) was used to assess stroke outcomes. Kaplan–Meier analysis was used to describe survival, and the Cox proportional hazards model was used to examine predictors of mortality.

**Results:**

A total of 135 patients were enrolled, with a mean age of 64.5 years. Hypertension was observed in 76%, and 20% were on regular anti-hypertensive medications. The overall 30-day mortality rate was 37%. Comparing patients with hemorrhagic and ischemic stroke, 25% had died by day 5 [25th percentile survival time (in days): 5 (95% CI: 2–14)] versus day 23 [25th percentile survival time (in days): 23 (95% CI: 11–30) (log-rank *p* < 0.001)], respectively. Aspiration pneumonia was the most common medical complication, occurring in 41.3% of patients. ECG abnormalities were observed in 54.6 and 46.9% of patients with hemorrhagic and ischemic stroke, respectively. The most common patterns were as follows: ST changes 29.6 vs. 30.9%, T-wave inversion 34.1 vs. 38.3%, and U-waves 18.2 vs. 1.2% in hemorrhagic and ischemic stroke, respectively. Independent predictors for case mortality were as follows: mRS score at presentation (4–5) [aHR 5.50 (95% CI: 2.02–15.04)], aspiration pneumonia [aHR 3.69 (95% CI: 1.71–13.69)], ECG abnormalities [aHR 2.28 (95% CI: 1.86–5.86)], and baseline stroke severity [aHR 1.09 (95% CI: 1.02–1.17)].

**Conclusion:**

Stroke is associated with a high 30-day mortality rate in Northwestern Tanzania. Concerted efforts are warranted in managing patients with stroke, with particular attention to individuals with severe strokes, ECG abnormalities, and swallowing difficulties to reduce early morbidity and mortality.

## Introduction

Stroke is the second leading cause of death and the third leading cause of death and disability-adjusted life years (DALYs) combined worldwide, according to the 2019 Global Burden of Disease report (1). In contrast to high-income countries (HIC), where there was a decrease in age-standardized stroke incidence, DALY, and mortality, low-middle-income countries (LMIC) had the highest age-standardized stroke-related mortality of more than 3.6 times (86%) with 87% DALYs (1). This decline in stroke morbidity and mortality in HIC reflects advancements in stroke management, leading to more favorable outcomes.

Countries in sub-Saharan Africa (SSA) have a high stroke burden and mortality (1): Stroke in Africa occurs at a younger age, which has significant socioeconomic implications (2–5). Previous hospital-based studies in Tanzania have reported varied 30-day case fatality rates, ranging from 30 to 60%, with a limited characterization of predictors of mortality (5, 6). In SSA, stroke severity, infections, elevated glucose levels, and fever are known predictors of 30-day mortality (5–7). There is an urgent need to find solutions to mitigate the rising number of strokes and the associated mortality in Tanzania. We aimed to investigate the predictors of 30-day mortality among patients with stroke admitted at a large tertiary teaching hospital in Tanzania.

## Materials and methods

### Study design and population

This cohort study was conducted at a tertiary teaching hospital, Bugando Medical Center (BMC), in Northwestern Tanzania. BMC offers specialized care to all in and outpatients from all over the country, particularly along the shores of Lake Victoria. Patients with stroke who met the World Health Organization (WHO) clinical definition were consecutively recruited between February 2022 and May 2022 after obtaining written informed consent from the patient, or next of kin (for those unable to consent due to stroke-related disabilities) (8).

### Data collection

Data were collected and managed using an electronic data-capturing database developed and hosted by VervigR. Information captured included baseline data, including gender, age, residency, marital status, level of educational achievement, and at least three available mobile numbers from the patient and next of kin. We also recorded any previous history or prescriptions for hypertension and diabetes mellitus and inquired about smoking and alcohol consumption.

#### Clinical measurement

Physical examination included assessment of the Glasgow coma scale score, temperature, pulse rate, and rhythm. Blood pressure readings were taken using a standard digital sphygmomanometer (Omron 10, Healthcare); three readings were taken 5-min apart. Hypertension was defined as systolic blood pressure (SBP) of ≥140 mmHg and/or diastolic blood pressure (DBP) of ≥90 mmHg or previous/current use of anti-hypertensive medications (9). Waist and hip circumference were measured using a tape measure and recorded in centimeters. The waist-hip ratio was computed and interpreted according to the WHO guidelines (10).

#### Laboratory investigations

We aseptically collected 15 ml of venous blood for complete blood count (Sysmex 1000 machine) and random total cholesterol (BIO-SYSTEMS machine). Capillary fingertip blood samples were collected to check for random blood glucose (RBG) levels and HIV rapid testing using a glucometer GLUCOPLUS^TM^ and SD Bioline, respectively. A fasting blood glucose (FBG) sample was collected the following morning for patients with RBG levels of ≥11.1 mmol/l. Diabetes mellitus diagnosis was defined as an RBG reading of ≥11.1 mmol/l or an FBG reading of ≥7 mmol/l. For patients who were HIV reactive to SD Bioline, confirmatory testing was performed using Unigold Biotech.

#### Medical complications

This included clinical seizures, infections: urinary tract infection and aspiration pneumonia (confirmed aspiration pneumonia or probable) (11), and the presence of bedsores (12, 13). Other complications included new onset fever lasting more than 24 h from an unknown source (13). Presumed venous thromboembolism was clinically diagnosed using Well's score (14). Miscellaneous complications were defined as any documented complication resulting in a specific medical or surgical intervention (e.g., gastrointestinal hemorrhage, constipation, episodes of cardiac failure, cardiac arrhythmias, and arthritis) (13).

#### Brain imaging

A non-contrasted brain computed tomography scan, acquired on a 128-slice CT Scanner (Siemens Somatom Perspective, Siemens Healthcare GmbH, Germany), was performed on all patients on admission, and images were interpreted by a neuro-radiologist. For ischemic stroke, the Alberta Stroke Program Early CT (ASPECTS) score was dichotomized to <7 and ≥7 and used for analysis (15). Hemorrhagic transformation was defined per European Cooperative Acute Stroke Study (ECASS II) (16). Midline shift was defined as any measurable shift of midline cerebral structures seen on axial view, specifically the septum pellucidum and/or the pineal gland (17). For those with hemorrhagic stroke, the location was documented, and the hematoma volume was measured using the modified ABC/2 method (18). We calculated the intracerebral hemorrhage (ICH) score with scores ranging from 0 to 5 (19).

#### Cardiovascular assessment

A 12-lead electrocardiography (ECG) (model ECG-3312B) was performed on all patients, and results were interpreted by a cardiologist for the presence of ST-segment depression or elevation, T-wave abnormalities, U-waves, and the presence of atrial fibrillation.

#### Stroke outcomes

Stroke severity was assessed using the National Institutes of Health Stroke Scale (NIHSS) on admission (20). Stroke outcomes were assessed using a modified Rankin scale (mRS) with scores ranging from 0 (no symptoms) to 6 (death) on admission up to 30 days (20). The date of death was recorded, and the time-to-event was computed using the date difference between the date of last contact (date of death or date of the last follow-up) and the date of symptom onset.

### Study variables

The dependent variable was case mortality, and the independent variables included were as follows: demographic data, medical co-morbidities and complications, stroke severity, stroke subtype, and ECG changes.

### Data analysis

Data analysis was done using STATA software version 15.0. Continuous variables were summarized and presented as means and standard deviation (SD) or medians and interquartile range [IQR]. Categorical variables were summarized as frequencies and proportions. Kaplan–Meier analysis was used to describe survival, where the 25th percentile survival time with respective 95% confidence intervals was estimated, and significant differences in survival times by stroke subtype were tested using the log-rank test. Crude and adjusted analyses were done using a Cox proportional hazards model. Hazard ratios (HRs), 95% confidence intervals (CIs), and corresponding *p*-values were obtained from the models adjusting for potential confounders. Factors for multivariable analyses were selected based on prior knowledge of the possible associations between stroke and mortality. These included age, sex, and previous history of stroke. Other variables with a *p*-value of <0.20 in the univariable model were included in the multivariable analysis, and a significance level was set as a *p*-value of <0.05.

## Results

### The proportion of strokes compared to the total medical admissions

**Figure 1:**
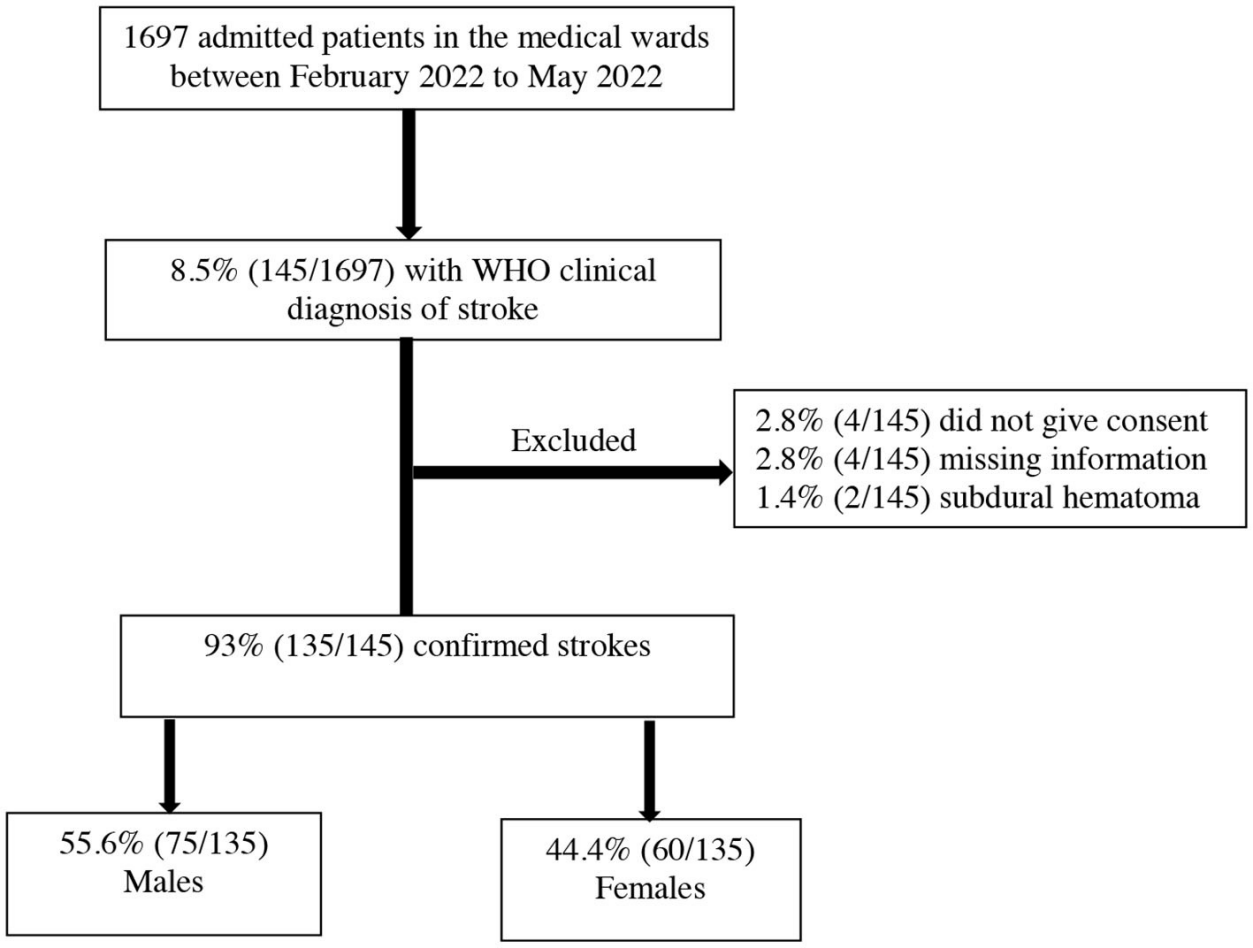
Recruitment flowchart.

During the study period from February 2022 to May 2022, there were 1,697 medical admissions. Out of these admissions, 8.5% (145/1697) met the WHO's clinical definition of stroke. We excluded 6.9% (10/145) patients for the following reasons: 2.8% (4/145) did not give consent to participate in the study, 2.8% (4/145) had missing information, and 1.38% (2/145) had subdural hematoma as summarized in Figure 1. The final proportion of patients with stroke compared to the total medical admissions was 7.9% (135/1697) (95% CI 6.7–9.4%).

### Demographic characteristics of the study patients

The mean age was 64.5 ± 14.7 years, and the majority were male patients, 55.6% (75/135). Notably, 57% (77/135) had secondary education or higher and 52.6% (71/135) were uninsured. The proportion of patients with a history of smoking or alcohol consumption was 28.9% (39/135) and 72.6% (98/135), respectively, as summarized in Table 1. The mean time from stroke symptom onset to hospital arrival was 2.67 ± 4.9 days.

**Table 1:** Demographic characteristics of the study patients.

**Variable**	**Frequency**	**Percentage**
**Age in years**		
< 60	52	38.5
≥60	83	61.5
Mean ± SD	64.5 ± 14.7	
**Sex**		
Female	60	44.4
Male	75	55.6
**Marital status**		
Ever married	132	97.7
Never married	3	2.2
**Education level**		
No education	34	25.2
Primary education	24	17.8
Secondary and above	77	57
**Insurance status**		
Insured	64	47.4
Not insured	71	52.6
**History of smoking**		
Never	96	71.1
Ever	39	28.9
**History of alcohol drinking**		
Never	37	27.4
Ever	98	72.6

A prior history of hypertension and diabetes mellitus was present in 76.3% (103/135) and 16.3% (22/135) of subjects, respectively. One-quarter of hypertensive (27/103) and one-half of patients with diabetes (11/22) were taking relevant regular medication. The mean time from stroke onset to brain imaging was 3.43 ± 3.8 days, and ischemic stroke occurred in two-thirds (81/135) of the patients. The median mRS and NIHSS scores on admission were 4 (IQR 3, 4) and 20.5 (17, 21), respectively. At presentation, 80.7% (109/135) were hypertensive and 3% (4/135) were HIV positive, as summarized in Table 2.

**Table 2:** Clinical characteristics of the study patients.

**Characteristic**	**Frequency**	**Percentage**
**Prior history of hypertension**	103	76.3
Not on regular medications	76	73.8
On regular medications	27	26.2
**Prior history of Diabetes mellitus**	22	16.3
Not on regular medications	11	50
On regular medications	11	50
**Atrial fibrillation**		
No	131	97
Yes	4	3
**HIV status**		
Negative	131	97
Positive	4	3
**Waist-hip ratio**		
Normal	83	61.5
Increased	52	38.5
**High blood pressure on admission (**≥**140/90 mmHg)**		
No	26	19
Yes	109	80.7
Stroke type^*^(***n*** = **125)**		
Hemorrhagic	44	35.2
Ischemic	81	64.8
**Functional status on admission**		
mRS 0–3	60	44.4
mRS 4–5	75	55.6
Median mRS (IQR)	4 (3, 4)	
**NIHSS score on admission**		
Median (IQR)	20.5 (17, 24)	
**Medical complications**		
No complications	72	53.3
Presence of complications	63	46.7

mRS, modified Rankin scale; NIHSS, National Institute of Health Stroke Scale.

* A total of 10 patients had a normal CT brain scan.

**Figure 2:**
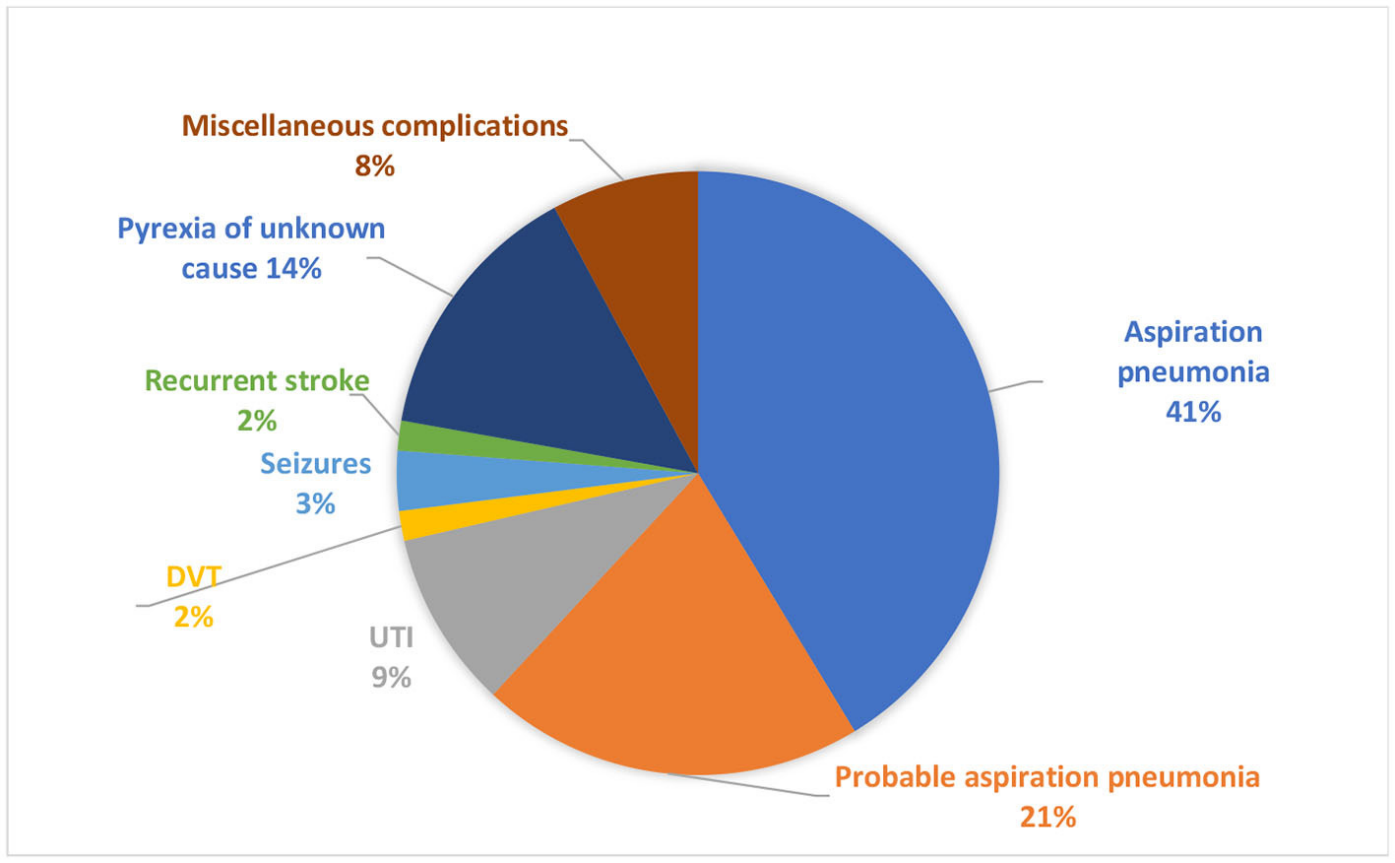
Medical complications post-stroke.

Post-stroke medical complications occurred in 46.7% (63/135) of patients. Confirmed aspiration pneumonia was the leading medical complication, seen in 41% (26/63), followed by probable aspiration pneumonia in 21% (13/63) and pyrexia of unknown cause in 14% (9/63) (Figure 2).

### Neuroimaging and ECG abnormalities among patients with hemorrhagic stroke

Neuroimaging and ECG features of the study patients with hemorrhagic stroke are presented in Table 3. A quarter (11/44) had a hemorrhage in multiple locations (both the cortical and subcortical regions), followed by the thalamus in 22.7% (10/44). In more than half (24/44), the volume of hematoma exceeded 30 ml and 63.6% (28/44) had an intraventricular extension. ECG abnormalities were present in 54.6% (24/44); the most common patterns were T-wave inversion in 34.1% (15/44), followed by ST segment changes in 29.6% (13/44).

**Table 3:** Neuroimaging and ECG abnormalities among patients with hemorrhagic stroke (*N* = 44).

**Characteristic**	**Frequency**	**Percentage**
**Hemorrhage location**		
Basal ganglia	9	20.4
Frontal lobe	2	4.6
Parietal lobe	3	6.8
Temporal lobe	6	13.6
Thalamus	10	22.7
Pontine	1	2.3
External capsule	2	4.6
Multiple locations	11	25.0
**Hemorrhage size (ml)**		
< 30	20	45.4
≥30	24	54.6
**Presence of intraventricular extension**		
No	16	36.4
Yes	28	63.6
**Intracerebral hemorrhage score (ICH)**		
< 3	24	54.6
≥3	20	45.4
**ECG abnormalities**		
No	20	45.4
Yes	24	54.6
**ST changes**		
No	31	70.4
Yes	13	29.6
**T-wave inversion**		
No	29	65.9
Yes	15	34.1
**U wave**		
No	36	81.8
Yes	8	18.2

### Neuroimaging and ECG abnormalities among patients with ischemic stroke

The majority were observed to have multi-territory infarction; 62.9% (51/81) and 53.1% (43/81) had midline shifts. ECG abnormalities were present in 46.9% (38/81); the most common patterns included T-wave inversion in 38.3% (31/81), followed by ST changes in 30.9% (25/81) (Table 4).

**Table 4:** Neuroimaging and ECG abnormalities among patients with ischemic stroke (*N* = 81).

**Characteristic**	**Frequency**	**Percentage**
**Infarct location (*****n*** = **81)**		
Occipital lobe	3	3.7
Basal ganglia	4	4.9
Frontal lobe	3	3.7
Parietal lobe	7	8.6
Temporal lobe	4	4.9
Thalamus	4	4.9
Pontine	2	2.5
External capsule	3	3.8
Multi-territory	51	62.9
**ASPECT score**		
< 7	40	55.6
≥7	32	44.4
**Midline shift**		
No	38	46.9
Yes	43	53.1
**Hemorrhagic transformation**		
No	69	85.2
Yes	12	15.8
**Hemorrhagic transformation subtypes** ^*^		
HI1	8	66.7
HI2	3	25
PH1	1	8.3
PH2	0	0
**ECG abnormalities**		
No	43	53.1
Yes	38	46.9
**ST changes**		
No	56	69.1
Yes	25	30.9
**T-wave inversion**		
No	50	61.7
Yes	31	38.3
**U wave**		
No	80	98.8
Yes	1	1.2

HI, hemorrhagic transformation; PH, parenchymal hematoma, ^*^N = 12.

### Survival experience and mortality by stroke subtype

**Figure 3:**
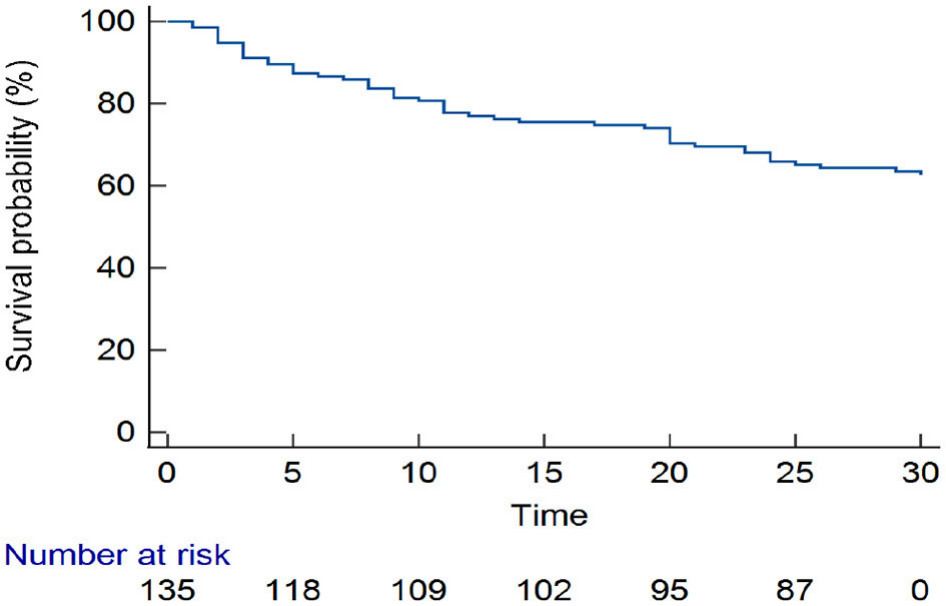
Kaplan–Meier curve estimating overall survival experience.

**Figure 4:**
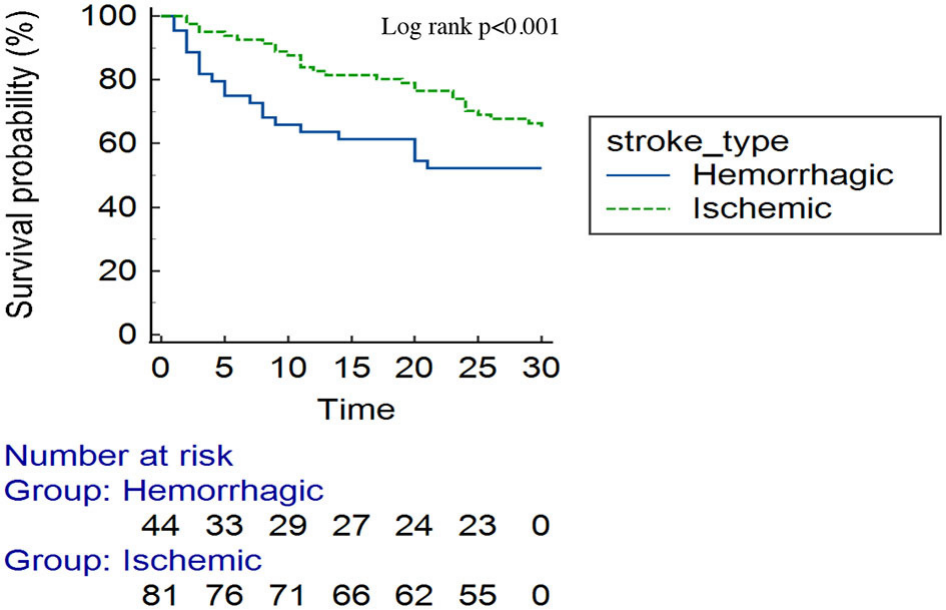
Kaplan–Meier survival curve by stroke subtype.

Approximately one-third (50/135) of the patients with stroke had died by 30-day follow-up. Overall, one-quarter of the patients had died by day 17 [25th percentile survival time (in days): 17 (95% CI: 9–24)] (Figure 3). Comparing patients with hemorrhagic and ischemic stroke, 25% of the patients had died by day 5 [25th percentile survival time (in days): 5 (95% CI: 2–14)] compared with day 23 [25th percentile survival time (in days): 23 (95% CI: 11–30)], respectively (log-rank *p* < 0.001) (Figure 4).

### Predictors of 30-day mortality

In the multivariable-adjusted analysis, independent predictors of 30-day mortality were NIHSS score on admission (30-day mortality increased by 9% for every unit increase in NIHSS score) [aHR 1.09 (95% CI: 1.02–1.17), *p* = 0.012], degree of disability on admission (mRS 4–5) [aHR 5.50 (95% CI: 2.02–15.04), *p* = 0.001], aspiration pneumonia [aHR 3.69 (95% CI: 1.51–13.6), *p* = 0.005], and ECG abnormalities [aHR 2.28 (95% CI: 1.86–5.86), *p* = 0.044] (Table 5).

**Table 5:** Predictors of 30-day mortality.

**Variable**	**HR (95%CI)**	***p*-value**	**aHR (95%CI)**	***p*-value**
**Age**				
< 60	1		1	
≥60	1.14 (0.64–2.05)	0.654	0.57 (0.22–1.50)	0.261
**Gender**				
Female	1		1	
Male	1.04 (0.60–1.82)	0.886	2.25 (0.83–6.05)	0.109
**Smoking**				
No	1			
Yes	1.10 (0.60–2.02)	0.756		
**Alcohol**				
No	1			
Yes	1.00 (0.54–1.84)	0.987		
**Waist-hip ratio**				
Normal	1			
Increased	1.05 (0.59–1.89)	0.859		
**BP on admission** ≥**140/90**				
No	1			
Yes	1.53 (0.69–3.40)	0.299		
**Fever**				
No	1			
Yes	1.18 (0.18–7.30)	0.861		
**mRS**				
mRS 0–3	1		1	
mRS 4–5	3.52 (1.80–6.89)	< 0.001	5.50 (2.02–15.04)	0.001
**Medical complications**				
No complications	1		1	
Aspiration pneumonia	13.95(6.84–28.45)	< 0.001	3.69 (1.51–13.69)	0.005
Others	1.91 (0.92–3.96)	0.081	1.15 (0.36–2.41)	0.815
**NIHSS on admission**	3.52 (1.80–6.89)	< 0.001	1.09 (1.02–1.18)	0.012
**Stroke subtype**				
Hemorrhagic	1		1	
Ischemic	0.59 (0.33–1.04)	0.066	1.61 (0.61–4.26)	0.335
**ECG abnormalities**				
No	1		1	
Yes	2.21 (1.24–3.95)	0.007	2.28 (1.86–5.86)	0.044
**Previous stroke**				
No	1		1	
Yes	0.65 (0.12–3.51)	0.622	0.43 (0.08–2.28)	0.335
**Hyperglycemia**				
No	1			
Yes	1.31 (0.53–3.24)	0.608		

## Discussion

In this study, we found that the 30-day post-stroke mortality rate was 37%. This mortality is high compared to mortality rates observed in HIC; for instance, the rate is 12.7% in patients admitted to Canadian stroke services (22). Furthermore, our mortality rate is higher than other comparable studies conducted in LMIC, for example, Nigeria, where the 30-day mortality rate was 23.8% (23). The differences in mortality could be attributed to the nature of the study design: The Nigerian study was a retrospective study of first-ever strokes, whereas this study was prospective and included all strokes (first and recurrent). Our findings are quite alarming and highlight the barriers associated with the management of acute stroke in a resource-limited setting. It has been well demonstrated that the presence of specialized stroke units, specialists, the use of intravenous thrombolytic agents, and endovascular procedures are highly effective at reducing morbidity and mortality from stroke (24). However, these services and resources are neither readily available nor affordable in SSA. In the current study, half of the patients had no health insurance coverage, which is a major barrier to accessing affordable, good-quality healthcare services for patients with stroke in Tanzania. A recent systematic review on health financing for universal health coverage in SSA reported that 27 out of 48 countries are affected by direct out-of-pocket payments for healthcare services of >30%. Therefore, the cost is likely to be a significant barrier to accessing healthcare, thus contributing to the high burden of preventable deaths (21).

In the present study, patients with hemorrhagic stroke had higher mortality than those with ischemic stroke, with one-quarter of patients dying by day 5 vs. 23. This is similar to global data, where the hemorrhagic stroke is associated with poor outcomes compared to ischemic stroke (25). Hypertensive hemorrhagic stroke results from the rupture of the thinner-walled perforating arteries, which supply the sub-cortical regions of the brain (26). Untreated, ongoing hypertension increases the risk of further bleeding, hematoma expansion, and intraventricular extension. In the present study, more than half (56.4%) had a hematoma size of ≥30 ml, and intraventricular extension was observed in 63.6% of patients. Similarly, elevated blood pressure readings were observed in more than two-thirds of patients (80.7%). Uncontrolled hypertension appears to be a major etiology for stroke, particularly the hemorrhagic stroke subtype in the current study, as almost one-half of the bleeds were located in the sub-cortical regions (particularly the basal ganglia and the thalamus), signifying a hypertensive etiology. Hypertension is a leading risk factor for stroke in Tanzania, and its early detection, treatment, and control cannot be overemphasized (5). This is a call for increasing community awareness for screening and treating hypertension in SSA. Furthermore, our findings also highlight the need for multidisciplinary stroke management on dedicated stroke units, with input from neurosurgeons for consideration of hematoma evacuation, decompressive surgery, or placement of an external ventricular drain.

Our study found that severe stroke, severe neurological impairment, aspiration pneumonia, and ECG abnormalities were independent predictors for 30-day mortality. High NIHSS and mRS scores are known predictors for 30-day mortality (27, 28). Notably, patients with stroke in the current study presented late to the hospital from the time of stroke onset (mean time of 2.67 days). This delay could be a major contributor to high admission NIHSS scores, stemming from a lack of community awareness of early recognition of stroke symptoms and signs and challenges in the overall healthcare infrastructure in Tanzania. Similarly, the relatively high admission NIHSS may also indicate that patients with milder strokes are not routinely accessing healthcare. Patients with severe neurological deficits have a high risk of medical complications such as aspiration pneumonia. This is in line with other studies and is a leading cause of early mortality in SSA (11). This is a call to advocate for specialized stroke units in Tanzania to help manage patients with stroke to prevent or reduce stroke-related complications.

Overall, ECG abnormalities were observed in more than one-third (46%) of the patients. The most common patterns in both stroke subtypes were as follows: ST changes (46.9 vs. 29.6%) and T-wave inversion (38.3 vs. 34.1%) for ischemic and hemorrhagic stroke, respectively, in line with previous studies (29). ECG abnormalities are a result of bidirectional interaction between the brain and the heart. This is thought to be caused by the over-activation of sympathetic activity and the hypothalamic–pituitary–adrenal axis, as well as immune and inflammatory responses resulting in brain dysregulation following an acute stroke. Similarly, the release of catecholamines causes vasoconstriction of peripheral and coronary vessels, which leads to myocardial ischemia (29). Therefore, there is a need for continuous electrocardiographic monitoring among patients with stroke to reduce early mortality.

Our study is limited by the following: It was a single center with a small sample size. Therefore, the results may not be generalizable. Most patients did not have a baseline ECG, so the changes observed might be attributed to other medical conditions. There may be other unmeasured or unknown confounders responsible for the observed data, including interdisciplinary and nursing input and timing of antithrombotic or anti-hypertensive therapy. We did not collect data on the final causes of death, which limits our ability to draw further inferences. Continuous ECG (telemetry) was not performed due to limited resources.

## Conclusion

In this present study, stroke is associated with a high 30-day mortality rate in Northwestern Tanzania. The hemorrhagic stroke subtype had the highest mortality, and independent predictors of death included higher NIHSS on admission with severe disabilities, aspiration pneumonia, and ECG abnormalities. Concerted efforts are warranted in the prevention and management of patients with stroke to reduce the associated morbidity and mortality.

## Data availability statement

The raw data supporting the conclusions of this article will be made available by the authors, without undue reservation.

## Ethics statement

The studies involving human participants were reviewed and approved by the joint Catholic University of Health and Allied Sciences-Bugando Medical Center Institutional Review Board approval number CREC/528/2022. The study was carried out in accordance with the tenets of the Declaration of Helsinki. The patients/participants provided their written informed consent to participate in this study.

## Author contributions

Conceptualization of the study: SSM, KM, FK, and GM. Data collection: GM, JS, LA, and JN. Interpretation of the results: GM, SSM, and PN. Data analysis: GM and SSM. Writing the initial manuscript: SSM. Critically reviewing and revising the manuscript: RA, FK, PN, BT, KK, RM, MM, FS, and KM. All authors read and approved the final manuscript.
